# Non-intrusive deep learning-based computational speech metrics with high-accuracy across a wide range of acoustic scenes

**DOI:** 10.1371/journal.pone.0278170

**Published:** 2022-11-28

**Authors:** Peter Udo Diehl, Leifur Thorbergsson, Yosef Singer, Vladislav Skripniuk, Annett Pudszuhn, Veit M. Hofmann, Elias Sprengel, Paul Meyer-Rachner

**Affiliations:** 1 Audatic, Berlin, Germany; 2 Charité–Universitätsmedizin Berlin, Corporate Member of Freie Universität Berlin, Humboldt-Universität zu Berlin, and Berlin Institute of Health, Department of Otorhinolaryngology, Head and Neck Surgery, Campus Benjamin Franklin, Berlin, Germany; Sejong University, REPUBLIC OF KOREA

## Abstract

Speech with high sound quality and little noise is central to many of our communication tools, including calls, video conferencing and hearing aids. While human ratings provide the best measure of sound quality, they are costly and time-intensive to gather, thus computational metrics are typically used instead. Here we present a non-intrusive, deep learning-based metric that takes only a sound sample as an input and returns ratings in three categories: overall quality, noise, and sound quality. This metric is available via a web API and is composed of a deep neural network ensemble with 5 networks that use either ResNet-26 architectures with STFT inputs or fully-connected networks with wav2vec features as inputs. The networks are trained and tested on over 1 million crowd-sourced human sound ratings across the three categories. Correlations of our metric with human ratings exceed or match other state-of-the-art metrics on 51 out of 56 benchmark scenes, while not requiring clean speech reference samples as opposed to metrics that are performing well on the other 5 scenes. The benchmark scenes represent a wide variety of acoustic environments and a large selection of post-processing methods that include classical methods (e.g. Wiener-filtering) and newer deep-learning methods.

## Introduction

Speech is one of the most important tools for communication, whether in person or via technology such as phone calls, online conferencing, podcasts, or virtual assistants. Improving speech understanding, especially in noisy settings, is the main goal of hearing aids and cochlear implants. These systems are constantly improving in areas such as speech enhancement, compression, speech-to-text and text-to-speech. However, these algorithms are prone to introducing artifacts and distortions to the speech that they process and would benefit from accurate estimates of human-perceived speech quality so that the quality of the system outputs may be evaluated.

Since this is a long-standing and widespread problem (e.g. telephone audio quality), the international standard by the International Telecommunication Union ITU P.835 [1] was established. In ITU P.835, the use of 3 metric categories is recommended: the quality of the speech signal, the amount of background noise, and an overall effect. The overall category takes both background noise and sound quality into account. The noise category describes the amount of (background) noise that is present in a sample and is typically the aspect that denoising systems try to reduce. The third category, sound quality, measures the quality of the audio, i.e., whether it’s free of distortions and processing artifacts, which is typically a key measure for text-to-speech systems. Ideally, estimates of sound quality and noise are decorrelated from each other, as there is often a tradeoff between these two aspects in applications. One example where this tradeoff is prominent, is speech enhancement, where aggressive denoising typically degrades sound quality and the preservation of sound quality often requires some background noise be retained. For all three of those categories, human ratings are the gold standard. Specifically, perceived speech quality is often estimated via subjective listening tests, in the form of mean opinion scores (MOS) [2], on a 1 (low) to 5 (high) scale, and then averaged to obtain one score per audio sample. However, human ratings are both costly and time consuming to gather, which makes it desirable to use computational metrics instead. Computational metrics can evaluate speech faster, in larger amounts, and cheaper than human raters.

Here we present a speech quality metric that is available for use via a web API https://metric.audatic.ai and addresses the most important aspects of such a metric: 1) it is non-intrusive (there is no need for a clean sound file as a reference), which allows it to be used on sounds recorded in real, noisy environments; 2) it evaluates the audio across the three ITU P.835 categories, predicting overall quality, amount of noise and sound quality; 3) its predictions across noise and sound quality are decorrelated from each other; and 4) it is useful across a wide variety of speech enhancement applications because it is trained on a large dataset of both unprocessed audio and audio processed by a many different processing algorithms,. The main contributions of the work presented here are, that we:

- outperform or match other speech metrics in 51 out of 56 benchmark scenes, where the metrics with results performing better use clean reference samples which we do not require
- created a large, labeled dataset of more than 1 million human ratings that contains more than 20 times more unique noisy speech samples than the next largest dataset that a metric was trained on
- improved the prediction by the deep neural networks through multiple means, such as input feature selection, specifically: a combination of wav2vec and STFT features, architecture selection, and ensembles
- do not require a clean reference since the metric networks are trained end-to-end on the sound sample inputs to human ratings, which is enabled by the large, labeled dataset
- make our metric easy and free to use via both a ‘drag & drop’ interface and web API at https://metric.audatic.ai.

In the following sections we describe related works (Related Works), explain in detail how our labeled dataset was gathered and how the models are trained (Materials & Methods), show how the model performs on a wide range of benchmark scenes and go into more detail on how computational demand scales with model performance (Results). Finally, we give more context about the results and our perspective (Conclusion). Supporting figures can be found in the S1 Appendix.

## Related works

Recent advances in deep learning have been applied to a variety of fields [3, 4], including advances in audio classification and processing [5–7]. Similarly, in acoustic scene classification where earlier models relied mostly on intelligent and sophisticated feature engineering [8, 9] are now utilizing deep learning in multiple applications without stringent computational requirements [10].

To date, a variety of computational metrics for speech and sound have been developed, including established ones such as PESQ [11], HASQI [12], STOI [13] which rely on feature engineering. While the first two were proposed to measure speech quality in different conditions, STOI intends to measure intelligibility. Most of these metrics are intrusive, i.e. they require a clean reference audio to estimate the difference between the processed and the reference/original audio sample. This implies, that they can only be used in simulated test environments where a clean-speech audio sample is available as a reference, and not in real-world environments where background noise is prevalent. Recently, metrics based on deep neural networks have been proposed to estimate speech quality [14–24]. Many of these use MOS ratings as training data and predict MOS scores. Some of these metrics are intrusive while others are not, and some predict only an overall score while others predict multiple score categories. The recently published DNSMOSP835 [25] (which is an extension of DNSMOS [14]) is a non-intrusive metric predicts the three ITU P.835 recommended score categories. It consists of a convolutional neural network trained on MOS ratings, and aims at evaluating modern speech enhancement algorithms. NISQA [15] is another non-intrusive convolutional neural network based model that is used to predict speech quality along five different dimensions that are relevant for modern voice over IP (VoIP) applications.

The main difference between our metric and other deep learning based metrics like DNSMOSP835 and NISQA is that ours is trained on a much larger number of human ratings and a wider array of sound types and processing methods. DNSMOSP835 is based on 600 unique noisy speech samples while we use over 14,000 unique noisy speech samples. This drastically improves generalization and accuracy of the predictions. The improvement in predictions makes our metric more attractive for development of new speech processing algorithms, such as enabling it to be used as a target for guided architecture searches of neural networks [7]. When comparing our results directly to other metrics such ESTOI, NISQA and DNSMOSP835, our metric shows improvements in the accuracy to human ratings across almost all evaluated categories and data sets. Note that a direct comparison to DNSMOSP835 is reasonable since it also relates to the IT P.835 standard but NISQA uses slightly different definitions of its categories and a direct comparison should therefore be treated accordingly.

## Materials & methods

**Fig 1:**
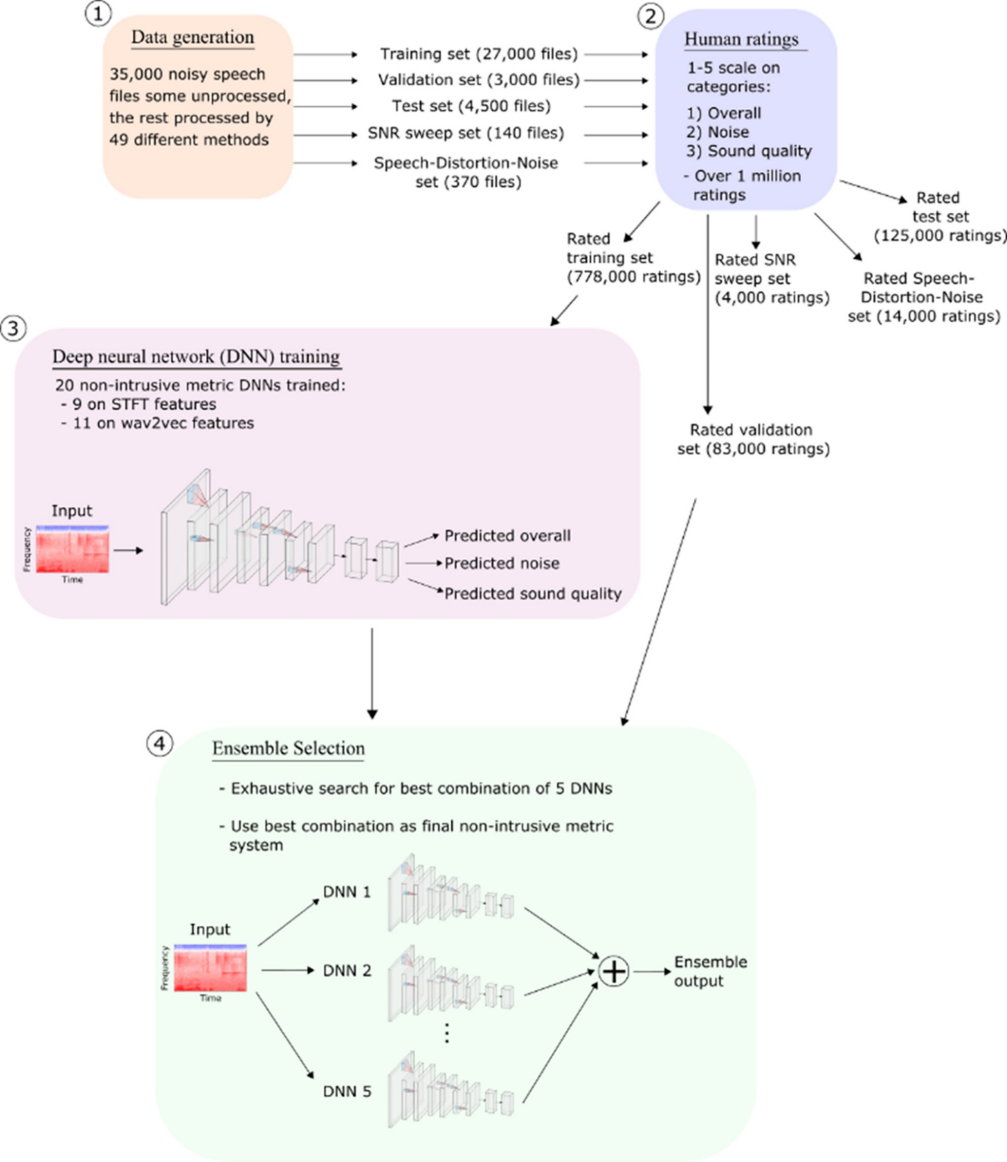
Training pipeline of the speech quality metric. Most data is generated by mixing speech and noise samples, other samples are real-world recordings. Each file is then rated approx. 27 times (9 times each for overall, noise, sound quality). The resulting rated training set is used to train 20 neural networks, 5 of which are then selected for the ensemble that is the final metric network used in the remainder.

Creating the presented deep learning based metric involved four stages: (1) data generation, (2) human rating collection, (3) neural network training, (4) ensemble selection (see Fig 1 for the corresponding numbers). In the first step of the data generation (1 in Fig 1), we created all datasets that are used to train our metric and compare it to human ratings and other metrics. The datasets are created by mixing speech from various speakers with various types of noise and then, for a subset of the sound files, post-processing them with a variety of processing methods (sub-section A, Data Generation). In order to train the metric and to compare results, we then gathered human ratings for those datasets (2 in Fig 1) using Amazon Mechanical Turk. The sound samples are rated on a scale of 1 to 5 for the three categories overall, noise, and sound quality (subsection B, Human Ratings). Those ratings are then used to train 20 deep neural networks end-to-end (3 in Fig 1), i.e. given a sound sample, the network has to correctly predict the average human rating. Those networks use two different types of input features: wav2vec embeddings [26] and short-time Fourier transform and corresponding also different architectures (see sub-section C, Deep Neural Network Architecture and Training). In order to achieve the best possible performance, we then pool the 5 best deep neural networks into an ensemble (4 in Fig 1). Since our application does not have highly stringent computing budget requirements, we use this to improve the correlations of our metric with the human ratings at the cost of computing time (subsection D, Deep Neural Network Training Ensemble Selection).

### A. Data generation

The goal was to create a deep learning based metric that is robust across a large variety of speakers, noise environments, signal-to-noise ratios (SNR) and methods of (post-) processing or denoising of the analyzed audio sample. Spanning such a large range of audio types required us to create a large, labelled dataset with sound samples for each of these categories. We divided this data into five subsets: training set (27694 samples), validation set (3078 samples), SNR sweep set (140 samples), Speech-Noise-Distortion set (370 samples), and test set (4500 samples).

**Fig 2:**
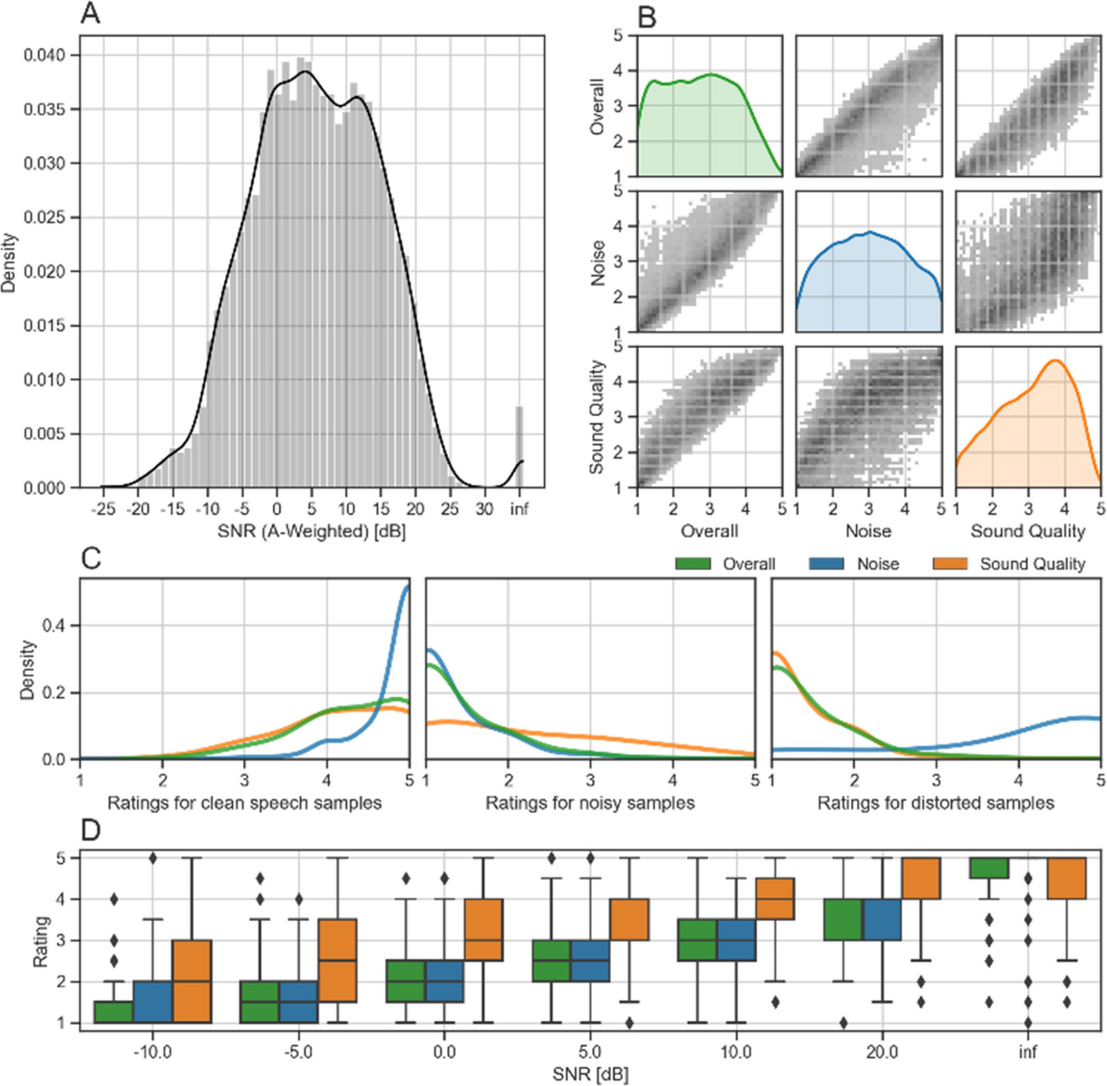
Data set and human ratings overview. A) SNR distribution of all unprocessed samples of the training set (n = 14847 samples). B) Human ratings for all sound samples (with and without processing) in the training set, SNR sweep set, Speech-Noise-Distortion set, and the validation set (n = 31282). C) Speech-Noise-Distortion data set ratings across categories (n = 126, n = 126, n = 118), sorted by sub-sets with clean speech, samples with large amounts of noise, and samples with strong distortions and artifacts. D) Ratings across the SNR range using the SNR sweep dataset (n = 20 samples per SNR, i.e. 140 samples in total).

The training set contains 14063 unprocessed samples (i.e. without any compression or denoising) created by mixing speech and noise samples, and 384 noisy speech samples from real-world recordings. The speech and noise samples are single channel snippets of 4 sec duration. Most of them are in English but a considerable amount are in other languages. These are then mixed and synthesized to create both single and multi-channel unprocessed samples. All mixed samples are between –20 and 30 dB SNR, with 1% set to be without noise (Fig 2A). We chose this distribution to resemble real-life noise environments [27] with a slight shift towards lower SNRs since at high SNRs scores would be too high to be reliably differentiated. Towards the lower SNR extreme ends, the speech is barely audible or not at all. -5 dB SNR could be a busy restaurant and 5 dB SNR could be a person talking next to you with a busy street at some distance in the background. Above 20 dB SNR the background noise is almost inaudible. We also included samples without noise (SNR is infinite) to include the corner cases. The samples are processed with one or multiple of 649 different models of 49 types, including a variety of deep learning based denoising methods, classical denoising methods (like beamformer), and explicitly added distortions. The resulting samples are always single-channel, since the multi-channel samples are converted to single-channel by their processing, e.g. beamforming. The validation set (not used for training) used for ensemble selection is generated in the same way as the training set.

The SNR sweep set (not used for training) contains only mixed speech and noise samples without any further processing. For given pairs of speech and noise, they are mixed with a range of SNR values ranging from -20 to infinity (only speech). This is useful to control the sound quality dimension (see definition below) as this should ideally only be impacted from adding noise when the noise directly interferes with the speech, e.g. when large amounts of white noise degrades the speech quality. However, the sound quality should not be impacted if an additional separate signal is partially overlapping, e.g. a siren or alarm.

The Speech-Noise-Distortion set (not used for training) contains 370 samples split into 3 categories: clean speech audio, very noisy audio that is not processed, and clean speech audio with strong processing artifacts. The processing augmentations included clipping, amplitude modulations, non-linear distortions and a comb filter. The resulting metric scores should be at the extreme ends of the individual categories of noise and sound quality. Lower scores for either noise or sound quality should also negatively impact the overall score, since both are required to be good for a good overall impression. We use these samples to test how correlated results of the metric are across these categories.

The test set (not used for training) contains 4500 sound samples and is constructed from the WHAMVox18 easy, WHAMVox hard (both freely available, including a detailed description of their construction), and the commonly used Valentini [28] set. We used 250 sound samples for each of the three data sets and processed them with 5 different methods, resulting in six (five processed, one unprocessed) times 750 sound samples. The 5 methods used were: 1) demucs [29] and 2) MHanet [30] (both current deep learning denoising systems), 3) SEGAN [31] (an older deep learning denoising system), 4) the dialogue isolate feature of the iZotope audio editing tool [32] (aimed at isolating speech), and 5) a Wiener filter [33] without particular fine-tuning. Those processing methods were not used in any other data set, to ensure a fair comparison between our metric and existing metrics.

### B. Human ratings

#### B.1. Human rating procedure

To obtain a reliable ground truth for each audio sample we crowdsourced ratings via Amazon Mechanical Turk. To ensure good consistency between the raters we provided detailed descriptions for each of the categories (overall quality, noise, and sound quality; Table 1), which are aligned with the international standard by the International Telecommunication Union ITU P.835 [1]. All categories are rated on a scale from 1 (worst) to 5 (best). Overall quality includes all aspects of a sound sample and should include all aspects of the sound file, be it artifacts in the speech, background noise or microphone quality. The noise category is focused on background noise and only refers to the amount of non-speech signal that is present. If the noise is loud enough to fully mask the speech, the sample should have a 1 as the noise rating, and if there is no background noise present, the noise rating should be a 5. The sound quality rating refers to the presents of distortions in the speech, artifacts (e.g. words cut off), or bad microphone quality. For example, a sample where many (parts of) words are chopped of that has no background noise should have a low sound quality score, a high noise score and a low to medium overall score, depending on the severity of the missing speech.

**Table 1:** Definition and rating instructions for human raters.

Score\ Category	Overall	Noise	Sound Quality
5 –Excellent	This sample has clear, easy to understand speech with no background noise and great sound quality.	There is no background noise at all.	Excellent sound quality (despite possible background noise). The voice sounds crisp and clear. Or no speech but high audio quality.
4—Good	The sound quality is good, the speech is easy to understand but there is a bit of background noise.	There is some background noise, but it is quiet and only slightly noticeable.	Good sound quality, but not excellent. Voice sounds a bit muffled, but still with good sound quality.
3—Fair	The speech is clear and intelligible. However, the intermittent background noise is quite distracting, and you need to concentrate to understand the speech.	There is a decent amount of background noise, but it is still relatively easy to understand the speech.	The microphone is of mediocre quality. Speech has some distortions.
2—Poor	This sample contains speech that is somewhat hard to understand, either due to intrusive background noise or distortions in the audio.	There is quite a bit of background noise in this sample and it’s almost masking the speech.	Voice sounds heavily distorted. Speech is modulated and sounds robotic.
1—Bad	The speech (if any is audible) is not understandable, due to very intrusive noise, loud babbling, and/or low sound quality.	This sample contains very intrusive noise. In addition, it seems to contain no speech.	While it might be possible to understand some words, the voice is very modulated and distorted. There are strong audio artifacts.

Human raters rated samples by category, first the overall quality for 50 samples, then noise for 50 samples and finally the sound quality for 50 samples. Audio samples are presented one at a time and raters are asked to rate them such that the ratings most closely align with the descriptions given in Table 1. To improve rating consistency, we also provided 2 sound examples per category for each of the three categories, i.e. 30 examples in total: 2 samples for each of the 3 categories, for each of the 5 scales. For each batch of samples.

We collected ratings from 342 individual raters, each of whom underwent a screening test to ensure high data quality. All raters were sourced from countries where English is a native language. The screening test consisted of two parts, was attempted by 1014 individual raters, and passed by 419 of them. Of those, 77 of the raters who passed the test did not complete more ratings after that, leading to the 342 final raters. The first part was a test to ensure that people were wearing headphones and was adapted from prior-art [34]. In the second part, participants rated approximately 10 samples for each of the three categories overall, noise and sound quality. The samples were chosen such that their rating is towards the extreme end of the scale, e.g. they either are of very good or very bad sound quality. For every sample, an acceptable range of ratings was defined. If a participant failed the headphone test, or too many ratings were outside the acceptable range, that participant was not allowed to provide more ratings. We did not directly test for hearing loss but assumed sufficient hearing since the screening test was passed. Screening ratings were not included in the dataset. Each sample is rated approx. nine times by different raters for the three categories, i.e. 27 ratings per sample. This results in a total of 777,642 ratings for the training set and 225,639 ratings for the various other sets (see Fig 1).

Since the ratings collected for the dataset were crowdsourced via Amazon Mechanical Turk, there was no direct contact with human participants as part of this study. We did not receive or collect any personal information of the raters. The raters and the authors gave consent to the Amazon Mechanical Turk guidelines, available under https://www.mturk.com/participation-agreement and https://www.mturk.com/legal-licenses.

#### B.2. Human ratings overview

To ensure that the human ratings match our definitions of the categories (Table 1), we analyzed the results (Fig 2B–2D) before training the neural network based metric on the data. One of the goals of the metric, and therefore for the underlying dataset, is that overall ratings, noise and sound quality indeed represent different aspects of the sound samples. A plot of the human ratings for each of those categories, both on the x-axis and the the y-axis (Fig 2B) visualizes the dependence of each category to the others. Examining their dependence shows that high overall scores imply high sound quality and noise ratings. However, high noise scores without high sound quality score (or the other way around) do not imply high overall scores, as desired. High noise ratings (i.e. low amounts of noise present in the samples) also do not imply high sound quality ratings, also as desired.

To further analyze how well the three rating categories are represented by the human ratings, we utilized the Speech-Noise-Distortion set, which contains 1) clean speech samples, 2) samples with noise but no distortions, 3) samples with no noise but distortions/artifacts. As the basic requirement, high quality sound samples should be consistently rated highly, which is confirmed by the ratings (Fig 2C, left). Almost all ratings of the clean speech samples are between 4.0 and 5.0, with most ratings being at 5.0, which is especially pronounced for the noise category, as desired. The noisy samples (Fig 2C, middle) have the lowest ratings in noise and overall, with a flatter distribution for sound quality. We would have expected/desired a slightly higher tilt towards higher sound quality ratings, as the sound quality is (according to definition) not impacted by noise. Analyzing the distorted samples (Fig 2C, right), they have low scores in the sound quality and overall category, while maintaining high scores on the noise category. This confirms that the sound quality category contains information that is distinct from the noise category, while still impacting the overall category (but not solely determining it).

Since the Speech-Noise-Distortion set analysis of the noisy samples revealed some dependence between the noise present in a sample and the corresponding sound quality ratings (Fig 2C, middle), we wanted to gain a better understanding of their relationship. To this end, we used the SNR sweep dataset (Fig 2D), which shows the SNR dependence for each of the categories. The overall and noise ratings have a strong linear relationship with the SNR, which is consistent with our definitions. Sound quality ratings have a higher variance, especially for low SNR samples, indicating that the raters agree less when sounds are in this SNR range. Since the data is unprocessed, according to our definition, the ratings should show mostly high sound quality (unless the recordings themselves happen to be of particularly bad quality), but the raters frequently do not rate the samples this way. We had multiple iterations to reduce this problem (including refining the definitions, rater screening etc.) but it is inherently difficult to judge for highly noisy samples whether there are also distortions or artifacts, which is why we accepted some deviation in lower SNRs. To minimize the impact of low-quality ratings, we periodically excluded unreliable raters (their previous data was still used for the data set).

### C. Deep neural network architecture and training

Our metric is composed of an ensemble of neural networks, trained on the labeled dataset described above. We trained 20 non-intrusive networks, using a mean-squared error loss on the difference of the human rating/label and the rating predicted by the network. Each network in the ensemble consists of an encoder from one of two possible architectures followed by several dense layers and a fully connected output layer predicting each of the three rated categories (overall quality, sound quality and noise). The first architecture uses a 2-dimensional convolutional encoder in the frequency domain, while the second uses a pretrained wav2vec 2.0 network to embed the raw waveform.

In the convolutional network, the waveform is first converted into the frequency domain using the short-time Fourier transform (STFT) with a Hann window with a size of 512 samples and a step size of 128 samples. The complex and imaginary parts of STFT are stacked along channel dimension. The resulting input tensor is of shape (B, 503, 257, 2), where B is the batch size. The input is then passed through a ResNet [35] consisting of 14 convolutional layers with 32 filters with kernel size 3x3, stride 1 and rectified linear unit (ReLU) activations. The convolution layers are followed by a global average pooling layer and 2 dense layers with ReLU activations and 256 and 128 units respectively followed by a dense output layer with 3 output units and a tanh nonlinearity.

In the wav2vec network we first pass the raw waveform through a pretrained wav2vec 2.0 network [26] with frozen weights, treating the non-quantized feature vectors produced by the last layer of transformer encoder of the wav2vec model as the embedding. The wav2vec embedding is then processed through 2 dense layers with ReLU activations and 256 and 128 units respectively followed by a global average pooling layer and finally a dense output layer with 3 output units and a tanh nonlinearity.

In total we trained 9 convolutional networks and 11 wav2vec networks. All networks from a given architecture were identical except for the random seeds used to initalize the weights at the start of training. The networks were trained for 350,000 (convolutional networks) and 90,000 (wav2vec) steps, with a batch size of 32, using an Adam optimizer with a learning rate of 0.0003. Throughout training, the mean-squared error between the human ratings and metric predictions declines until training reaches a plateau (Fig A1 in S1 Appendix).

### D. Deep neural network ensemble

#### D.1. Deep neural network ensemble selection

To improve the accuracy of the predicted ratings, we pooled multiple neural networks into an ensemble, where the final score is the average output from the networks in the ensemble.

Using the 20 total trained networks for creating an ensemble, we only considered ensembles that would be within an acceptable computational budget. The chosen threshold here is to run within 1 second per 4 second sample on an AMD Ryzen Threadripper 1920X 12-Core CPU which was limited to using 6 cores for processing. We then chose the ensemble that had the best combined performance on our validation set, noting that adding more models had diminishing returns in performance.

#### D.2. Deep neural network execution

The entire pipeline is implemented in Python 3.8, relying heavily on TensorFlow 2.4.1 for implementing the deep learning parts and Pandas for the data pipeline.

## Results

### A. Metric performance evaluation

**Fig 3:**
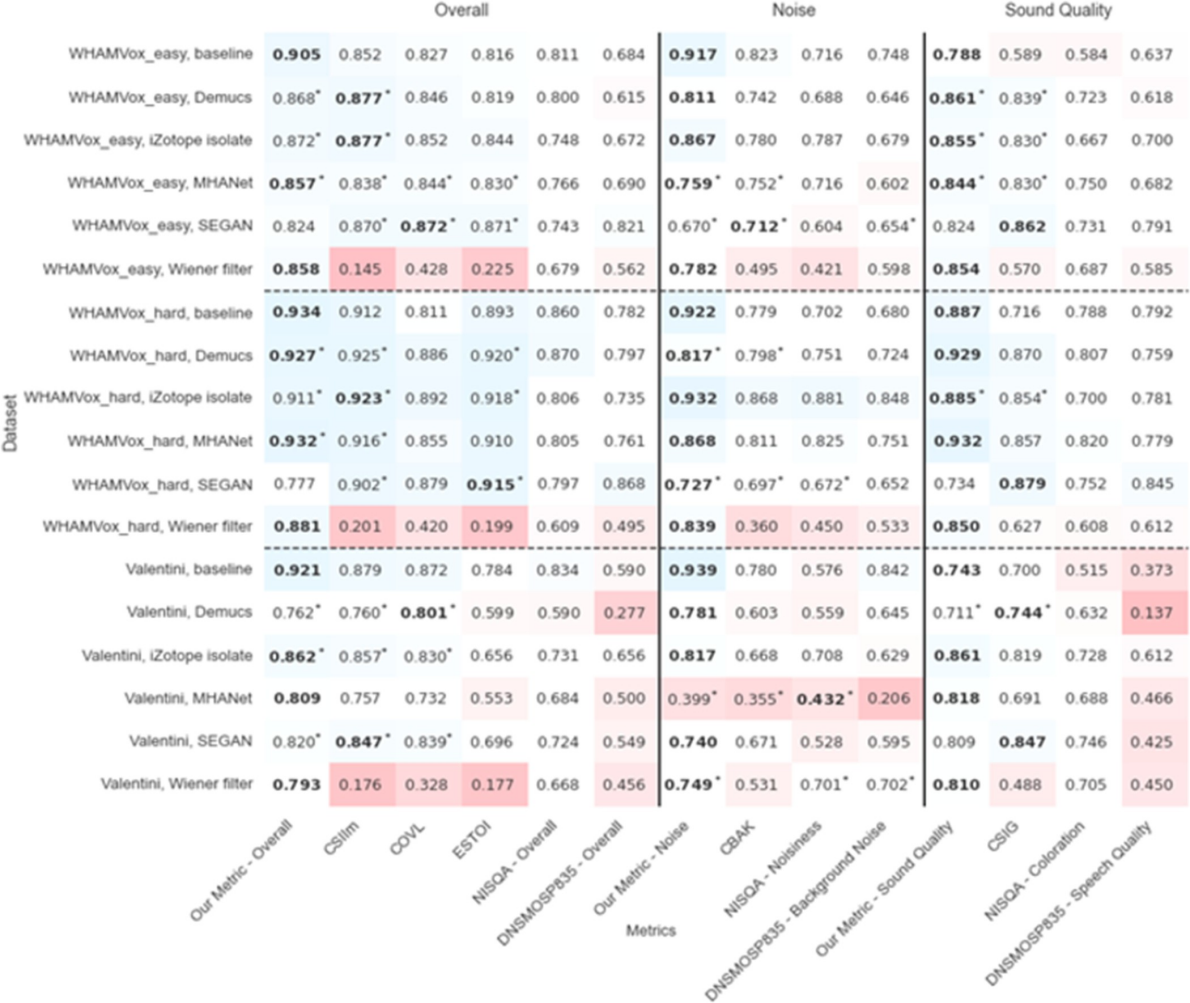
Pearson correlations of intrusive and non-intrusive metrics with human ratings on overall, noise, sound quality. Ratings are subsets of the WHAMVox_easy set (n = 250), WHAMVox_hard (n = 250) and of the Valentini set (n = 250). Red shades indicate low correlations and blue shades high correlations. The numbers in bold are the highest correlations in each category and the numbers with an asterisk * have no statistically significant difference in correlation [36] to the highest based on the t-test.

The central requirement for a good computational speech metric is approximating human psychoacoustics well. The first columns for overall, noise, and sound quality in Fig 3 show Pearson correlations of our metric with human ratings. Correlations are measured on a hold-out test set that was rated by human raters, too and consists of different speakers, different environments and different noises to what our metric was trained on. Correlations up to 0.4 are too poor to be useful for most applications. Between 0.4 and 0.7 they could be use to make out a coarse trend in the quality but for correlations above 0.7, the corresponding metric can be considered useful for guiding algorithmic development. However, higher correlations are better, as some applications rely on precise feedback, e.g. guiding a neural network architecture search or even training a neural network directly on the rating predictions provided by the metric. Most of the correlations of our metric are between 0.8 and 0.9, which is more than sufficiently high to use the presented metric as a substitute for human ratings in many scenarios.

To compare the established intrusive metrics (CSIIm, COVL, ESTOI, CBAK, CSIG) and new non-intrusive deep learning based metrics (DNSMOSP835 [14] and NISQA [15]) to ours, we use a test set of samples with a variety of speech, noise and processing methods (Fig 3). We neither trained our metric on the speech or noise samples, nor on the processing methods used in the test set. In 51 out of 56 tests, our metric outperformed or had no statistically significant difference (using the t-test) to the best performing metric in each benchmark. In 54 out of 56 tests, our metric either had the highest correlation or was not significantly lower than the metric with the highest correlation compared to the other two deep learning metrics. The established metrics ESTOI [37] and COVL [38] are mostly capable of capturing the overall human ratings of the sound samples, while CBAK [38] and CSIG [38] mostly capture the noise and sound quality categories well, respectively. Note that our metric can estimate ratings better than those traditional metrics, without requiring a clean reference sample. In applications where a clean reference sample is available and the computing budget is highly limited, intrusive metrics such as CSIIm, CBAK, CSIG can provide a fast and reasonably accurate solution. The five out of 56 categories where our metric performed worse were tests using the SEGAN model for processing which creates an unusual type of artifact that non-intrusive metrics could not judge well. In all of the five tests higher correlations were achieved by traditional intrusive metrics (COVL and ESTOI) since they rely on a clean speech sample to better assess the actual speech quality. The two deep learning based metrics DNSMOSP835 and NISQA show mixed results and for overall and sound quality correlation scores they were significantly worse than the best performing metrics, on all 36 tests. In the noise category they were able to achieve 2 and 3 out of 18 correlations with human ratings that were not significantly different from our metric, respectively for DNSMOSP835 and NISQA. When comparing mean-squared errors (MSE) between the metric predictions and human ratings all show the same results for the best prediction and our metric outperformed or matched all other metrics on 52 out of 56 benchmarks (Fig A3 in S1 Appendix). Similar to the Pearson correlations, the other deep learning metrics NISQA and DNSMOSP835 showed significantly higher MSE in all 36 overall and sound quality benchmarks, and in 4 of the 18 noise benchmark scenes their MSEs were not significantly different from the MSE of our metric. Another way of comparing the metric results with human ratings are residual error distributions [36], here with a threshold of 0.4 (Fig A4 in S1 Appendix). Also, this analysis confirms the robust results of our metric, since it outperformed or matched all other metrics on 54 out of 56 benchmarks. Both NISQA and DNSMOSP835 showed particularly low correlations for the Valentini data set and Wiener filter processing. Possible explanations could be overfitting to a particular type of processing or dataset, or insufficient training samples on those types of audio. Note that our training set contains almost 15,000 unique noisy speech samples while e.g. DNSMOSP835 only uses 600 different noisy speech samples that are processed in different ways (see Fig A2 in S1 Appendix for the dependence of our metric on the fraction of samples used).

Note that prior to evaluation, each metric is normalized with a 3rd order mapping [36]. This is done since the score distribution of each MOS estimator tends to match that of its training MOS dataset, and those can have varying distributions based on how the ratings are collected. For example, if a MOS experiment contains mostly high quality samples, then raters will tend to score them lower than they would in a more balanced experiment (thinking they should use the whole range). Therefore, we follow the recommendation in ITU-T P.1401 [36] and apply a 3rd order mapping between predicted scores of an estimator and the MOS ratings, before evaluating the performance of the estimator. The mapping has the form f(y) = a + by + cy^2 + dy^3, where y are the raw predictions, and parameters a-d are set to minimize the RMSE between the MOS ratings and the mapped predictions, while being monotonously increasing over the range of raw predictions. One can think of this as an adaptation of an estimator to an MOS dataset that differs from its training MOS dataset, and this results in a fairer comparison between estimators.

**Fig 4:**
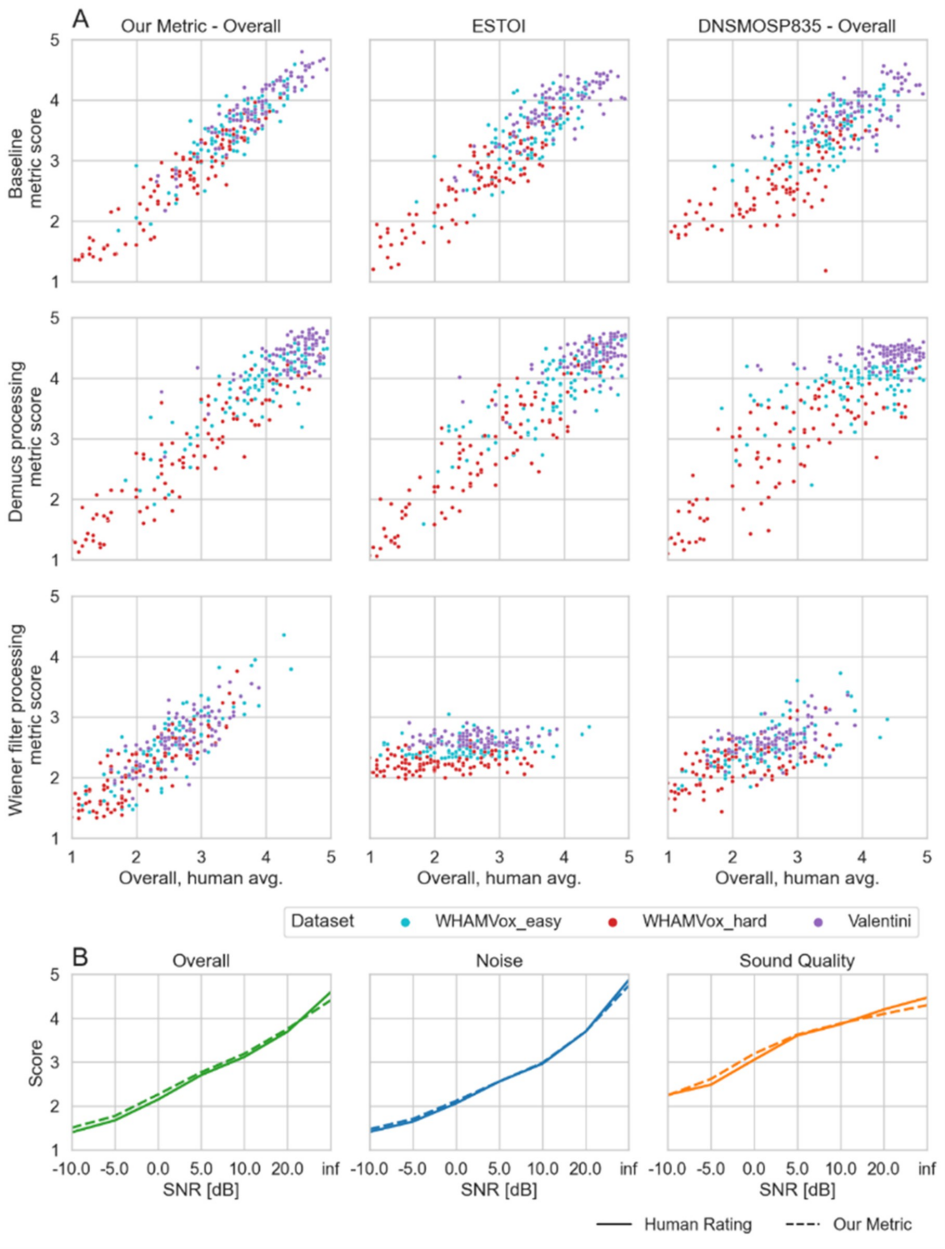
Comparison of our non-intrusive metric with ESTOI (intrusive) and DNSMOS P.835 (non-intrusive) on human ratings on overall, noise, sound quality and comparison of human ratings with our metric. A) The first row shows audio without processing, the second row shows audio processed with the deep learning-based denoising system demucs, and the third row shows audio processed with a Wiener filter. The plots are normalized to account for the individual differences [36] in the datasets (a comparison of the graphs without corrections is included in the appendix). B) Comparison of the average human ratings with our metric for the SNR sweep data set (n = 140) over an SNR range from -20 dB to clean audio, across the categories overall, noise, sound quality.

To better understand metric differences and failure cases, we show scatterplots of the overall category in selected datasets and processing methods (Fig 4A with normalization, Fig A5 in the S1 Appendix without normalization). In the first row of samples without any processing (Fig 4A, baseline), all shown metrics, ours, ESTOI and DNSMOSP835, show the expected error pattern, estimate the trend correctly and mainly differ by their precision in this. When processing the samples with the deep learning based denoising system demucs (Fig 4A, second row), both our metric and ESTOI predict the ratings fairly well. DNSMOSP835, also work well for the WHAMVox sets but not for Valentini, where it degenerates to a mostly uniform rating. In our experience, this can happen when the metrics are trained either with too few examples with high overall quality, or when trained with too few medium-quality samples that are then processed by a strong denoising system. The third row of samples that are processed using a Wiener filter clearly illustrates the advantages of using deep learning based metrics. The Wiener filter was intended to simulate a failed experiment where the creators are trying to optimize a denoising system but did not fine-tune the filter well, resulting in an underwater-like noise. Both deep learning based metrics were able to differentiate samples of different qualities, while ESTOI degenerated to a uniform rating. Note that the difference in average estimates between the different dataset with Wiener filter on ESTOI sets is due to the normalization we applied. Overall, the metrics seem to handle failure cases gracefully in that they degenerate to a small range of values, which can be detected in real-world scenarios.

Next, we want to confirm the broad applicability of our presented metric across a wide range of SNRs. A comparison of human ratings and ratings predicted by our metric on the SNR sweep set (Fig 4B) shows that the ratings are well correlated with correlations of 0.975, 0.978 and 0.929 for the overall, noise, and sound quality categories, respectively. The main inconsistencies occur for extreme sounds–clean or almost purely noise, but even there they stay within a score difference of 0.2. Compared to human ratings, our metrics network shows a slight bias towards the average ratings and tends to avoid more extreme ratings that are close to 1 or 5.

**Fig 5:**
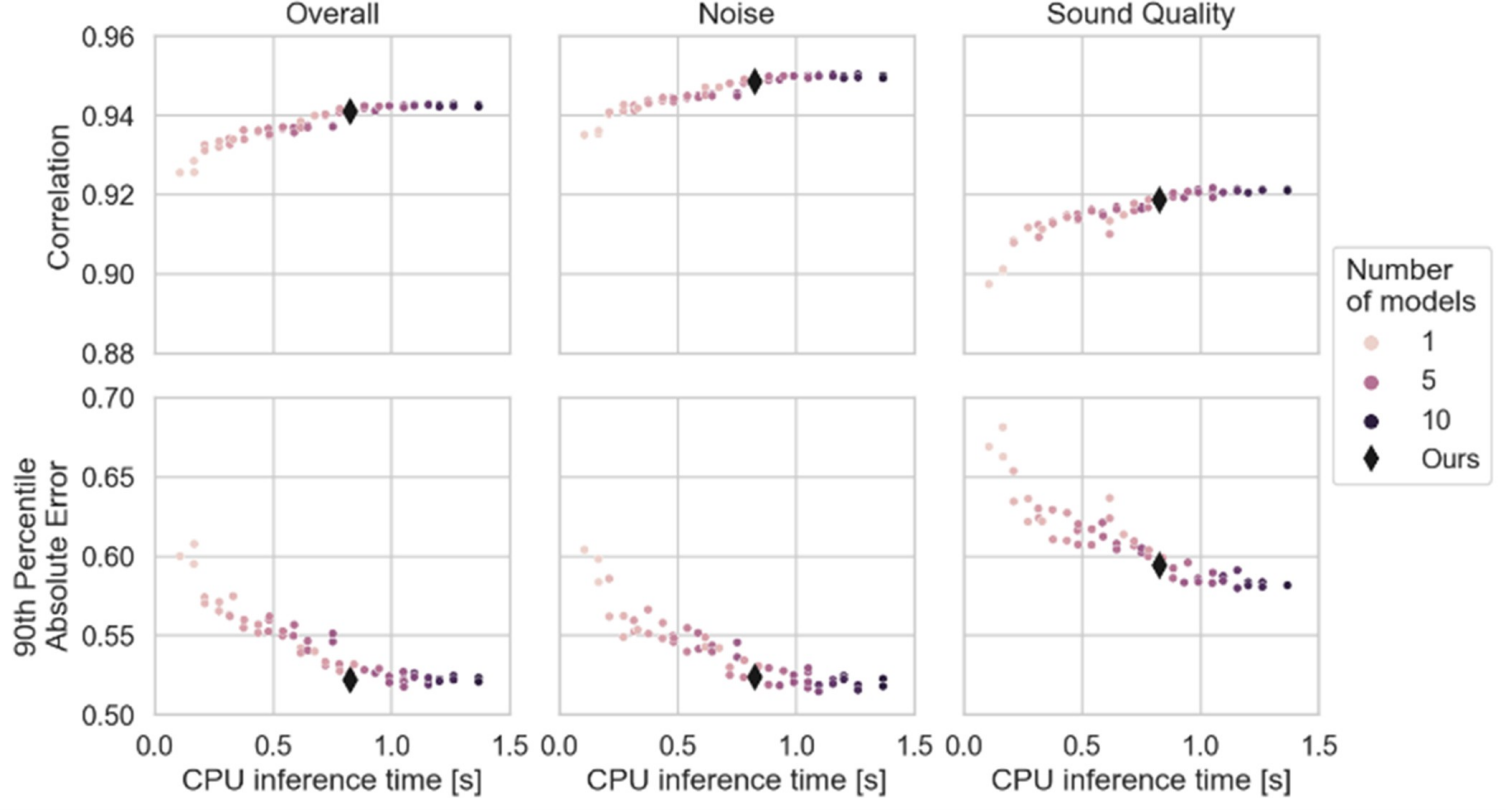
Ensemble size performance vs. computational complexity. Computation times are based on the validation set (n = 3078 samples) calculated on an AMD Ryzen Threadripper 1920X 12-Core CPU but artificially limited to using 6 of those cores.

Additionally, to scaling well with the large dataset size (see S1 Appendix) we can further improve correlations with human ratings by basing our metric on an ensemble of deep learning models. Creating ensembles instead of using single models is expected to lead to better performance at the cost of computational complexity/processing time. To explore the tradeoff between accuracy and computational demand, we compared single neural networks and a range of ensembles that consist of two to ten neural networks (Fig 5). The best single model has an overall correlation of 0.928 with human ratings on the validation set, while the best ensemble has a correlation of 0.943, i.e. using an ensemble improves correlations by 1% - 2% compared to the best individual models. Our target for the metric is to be useful for evaluating systems that modify speech (e.g. speech denoising) and while it has to be executed fast enough to evaluate thousands of speech enhancement models, e.g. for an evolutionary search [7], the computational budgets for this use-case are sufficient to allow for ensembles instead of a single model. Therefore, we chose an ensemble that simultaneously exhibits high correlations at 0.941 and low 90^th^ percentile absolute error, with the 90th percentile absolute error being the 90th percentile value of the per file absolute errors. It contains 5 networks, with 3 using STFT inputs and 2 using wav2vec inputs. The ensemble captures the performance of our model on the more challenging examples of the dataset, similar to a lower bound on performance. While optimizing both correlations and absolute error, we are also choosing the ensemble to have low a computing complexity with 0.8 seconds of execution time per 4 second sample on an AMD Ryzen Threadripper 1920X 12-Core CPU which was limited to using 6 cores for processing to enable more tasks in parallel (70ms on an NVIDIA 2080 RTX). The computational load renders it sufficient to be used for tightly spaced algorithm evaluation on small to medium sized data sets, e.g. as evaluation during neural network training.

### B. Web API

To further speed up research in speech-quality sensitive areas such as speech enhancement or compression, we are making our metric freely available online via a web-based drag & drop interface and via a web API, both under metric.audatic.ai.

Using the web-based metric does not require any computational resources from the user since the computation is performed by the server. Multiple files (up to 15) can be uploaded at once and all files will be added to the queue of files to be evaluated. After the evaluation finished, their corresponding overall, noise and sound quality scores can be queried by the user. To prevent single users from monopolizing the API, there is a limit of 1 hour of audio files that can be submitted per user per 24 hours. A detailed explanation of the API usage can be found in the appendix and under metric.audatic.ai.

We encourage users to experiment with a variety of samples and report cases where ratings differ from the user’s opinion to the authors. We will use this feedback to continue to improve the metrics and continually increase its accuracy.

## Conclusion

The presented speech metric is non-intrusive (does not require clean speech reference samples), predicts ratings across three categories representing overall impression, noise, and sound quality, and has a high correlation with human ratings across all three. We make it publicly available for use via our web API and a simple drag & drop interface at https://metric.audatic.ai, without requiring computational resources from the user.

Our non-intrusive estimator is especially useful in settings where it is difficult, expensive, or impossible to obtain clean speech samples as references. This enables the metric to be used to analyze performance of algorithms in real-world scenarios where no clean samples are available, including data collected from phone calls or video conferences. Additionally, the fact that the estimator predicts decorrelated ratings for noise and sound quality categories enables users to optimize algorithms towards specific properties or certain user groups. For example, a speech enhancement algorithm targeted at improving speech intelligibility for hearing impaired listeners might aim to maximally reduce noise given a certain acceptable level of artifacts (sound quality), while one targeted at normal hearing users of a teleconferencing system might aim to maximize sound quality given an acceptable level of background noise.

The presented metric also enables training of larger speech-based systems with direct feedback on the three categories overall, noise, and sound quality [7]. As the main requirement to enable this, the speech metric must represent human scoring very well, to avoid misguiding the training. Also, given the large data corpuses speech systems are trained on (often hundreds or thousands of hours of speech), speech metrics used to guide the training process have to be highly robust to a variety of speech types and environments, which is what deep learning based metrics allow for. In extreme cases, without this robustness it could even lead to a situation where the speech system (e.g. speech compression) learns to do well on the speech metric but not on many real-life situations, similar to adversarial training [39]. In practice, this can often be avoided by mixing different losses or training target, like a traditional mean-squared error with a more informative and targeted measure such as a speech metric. Future implementations of metrics such as ours could be further refined to better suit particular applications. For example, when developing speech enhancement algorithms for hearing impaired listeners, the metric could be fully trained or finetuned on ratings collected from hearing impaired listeners. Similarly, if the use-case of the metric is to assess video calls, the metric could be finetuned using ratings obtained from calls recorded on the desired call platform.

Our metric improves upon other investigated metrics in the precision with which it can match human ratings and in the types of samples it can handle. This is especially prominent for sounds processed by the classical Wiener filter, where most other metrics (both deep learning and non-deep learning based) failed to replicate human ratings well. While non-deep learning based metrics continue to perform on par with deep learning based approaches in many settings, it is more difficult to improve upon their failure cases. This is in contrast to deep learning based metrics, where a common way to fix failure cases is to include more samples in the training set that are similar to these failure cases. Continuing to improve upon these failure cases should allow deep learning based metrics to approach human-level performance for ratings of any class of samples. When designing our metric, we took great care to span a large variety of sample types and to train the metric to represent human ratings well on all of these. Therefore, due to the flexibility and scalability of deep learning based metrics, we expect the field to move even more towards such end-to-end trained metrics and replacing traditional hand-designed metrics.

Comparing results of our presented speech metric with the other two high-performing deep learning based metrics NISQA and DNSMOSP835, we have higher or not significantly different correlations than both of them on 54 out of 56 benchmark scenes across the categories overall, noise, and sound quality. In the 36 benchmarks for overall and sound quality, NISQA and DNSMOSP835 had significantly lower correlations than the best metrics. However, NISQA used a different definition for the sound quality category which might impact the results in those 18 benchmark scenes. Other analyses using mean-squared error and thresholds of residual errors also show outperforming or matching results of our metric in 54 out of 56 tests compared to NISQA and DNSMOSP835. All three measures of fidelity (correlations, mean-squared error and thresholds of residual errors) favor the same metrics in the tests, indicating a high robustness of the shown results.

Additionally, the other deep learning metrics NISQA and DNSMOSP835 are not publicly available for use, limiting their impact on the development of other speech systems. Nevertheless, we expect a broader trend of the field to shift towards deep learning based speech metrics, especially subfields like the development of systems for hearing impaired or video/phone call systems where fine-tuned metrics can represent the targeted application better.

In summary:

- non-intrusive metrics such as ours are now better or as good as the traditionally used metrics, combining high accuracy with general applicability
- increases in accuracy and generalization enable usage of deep learning speech metrics for training of other speech-based systems like speech synthesis, compression, or denoising
- deep learning based speech metrics have clear paths to further improve, e.g. fine-tuning to applications like hearing loss or phone calls, or fixing possible remaining error cases by improving the underlying training data set.

We hope that our metric and its freely available web API will be of service to the community, and we hope for ongoing feedback, so we can continue to improve upon the remarkable progress in this field.

## Supporting information

S1 Appendix (DOCX)
