# Supplementary material for: Non-intrusive deep learning-based computational speech metrics with high-accuracy across a wide range of acoustic scenes
Source: PLoS One. 2022 Nov 28;17(11):e0278170. doi: 10.1371/journal.pone.0278170 (PMC9704549; doi:10.1371/journal.pone.0278170)
Supplement: S1 Appendix — (DOCX) [file pone.0278170.s001.docx]

Appendix

###
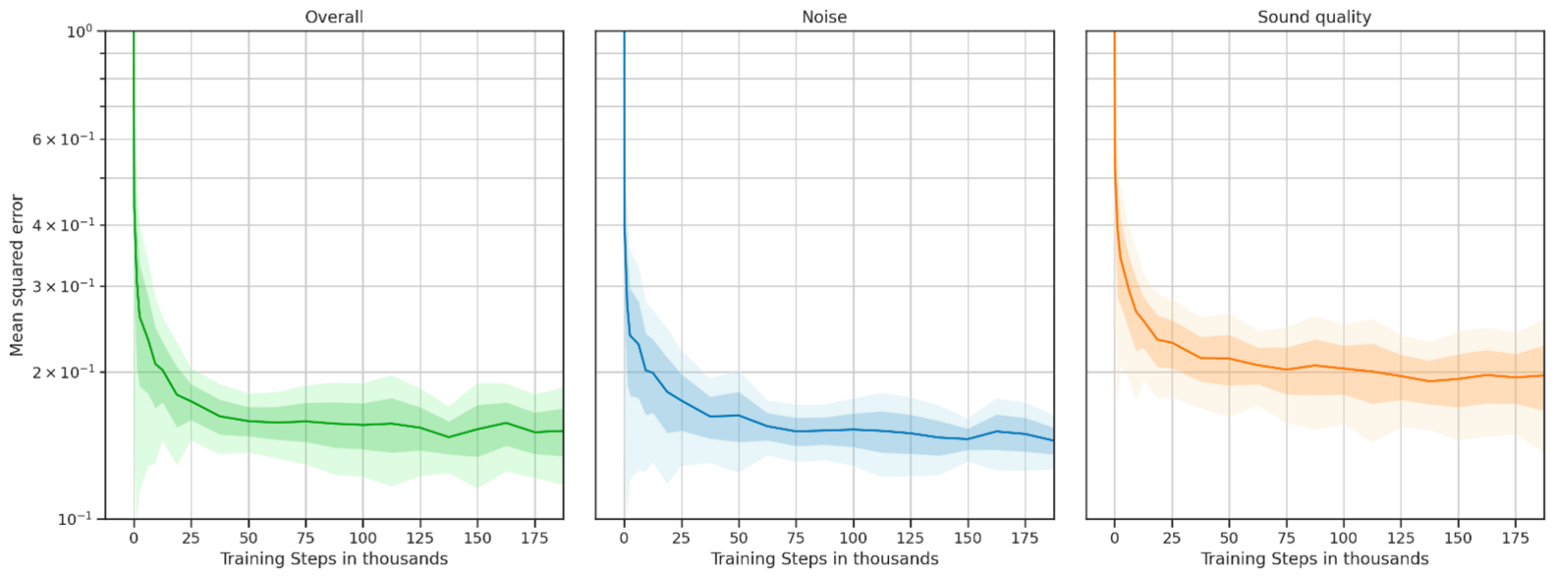


### Fig. A1. Average mean-squared error (MSE) between human ratings and metric predictions during training of the 20 deep neural networks. The dark shaded area denotes the standard deviation of the MSE and the lighter shaded area two standard deviations of the MSE.

###
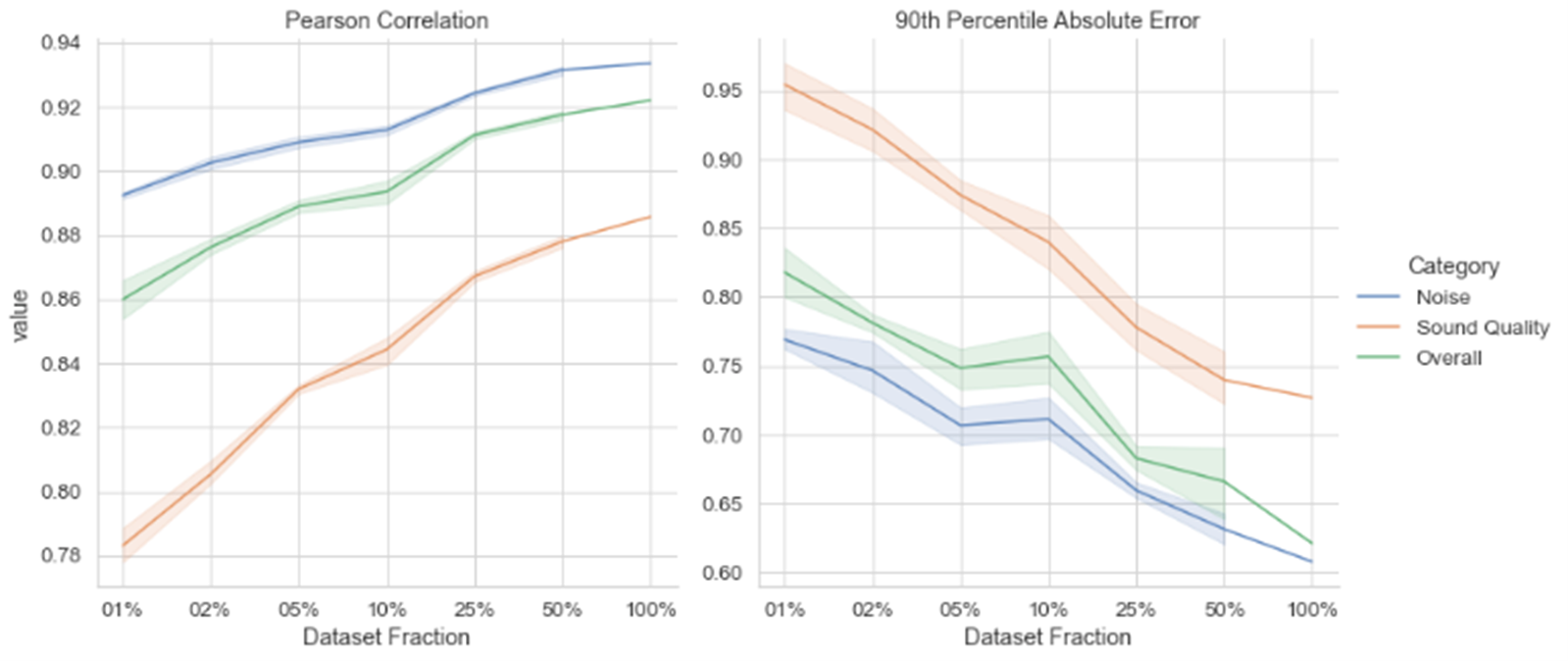


### Fig. A2. Dependence of the correlation and absolute error on dataset size between our metric and the human ratings.


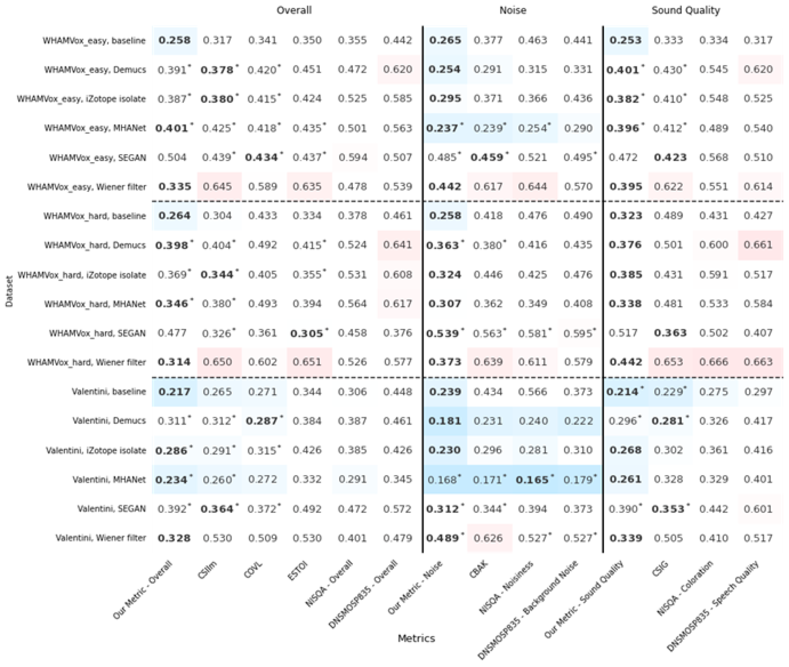


**Fig. A3.** Mean-squared error of intrusive and non-intrusive metrics with human ratings on overall, noise, sound quality. Ratings are subsets of the WHAMVox_easy set (n=250), WHAMVox_hard (n=250) and of the Valentini set (n=250). Red shades indicate low correlations and blue shades high correlations. The numbers in bold are the highest correlations in each category and the numbers with an asterisk * have no statistically significant difference in correlation to the highest based on the f-test.


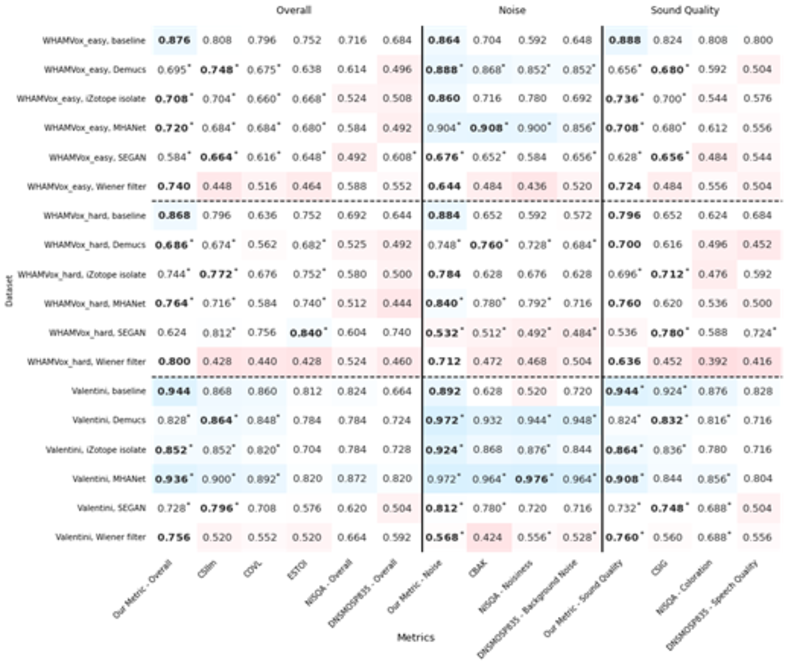


**Fig. A4.** Thresholds of residual error distributions [36] of intrusive and non-intrusive metrics with human ratings on overall, noise, sound quality. The chosen threshold is 0.4. Ratings are subsets of the WHAMVox_easy set (n=250), WHAMVox_hard (n=250) and of the Valentini set (n=250). Red shades indicate low correlations and blue shades high correlations. The numbers in bold are the highest correlations in each category and the numbers with an asterisk * have no statistically significant difference in correlation to the highest based on the 2-sample z-test of proportions.

###
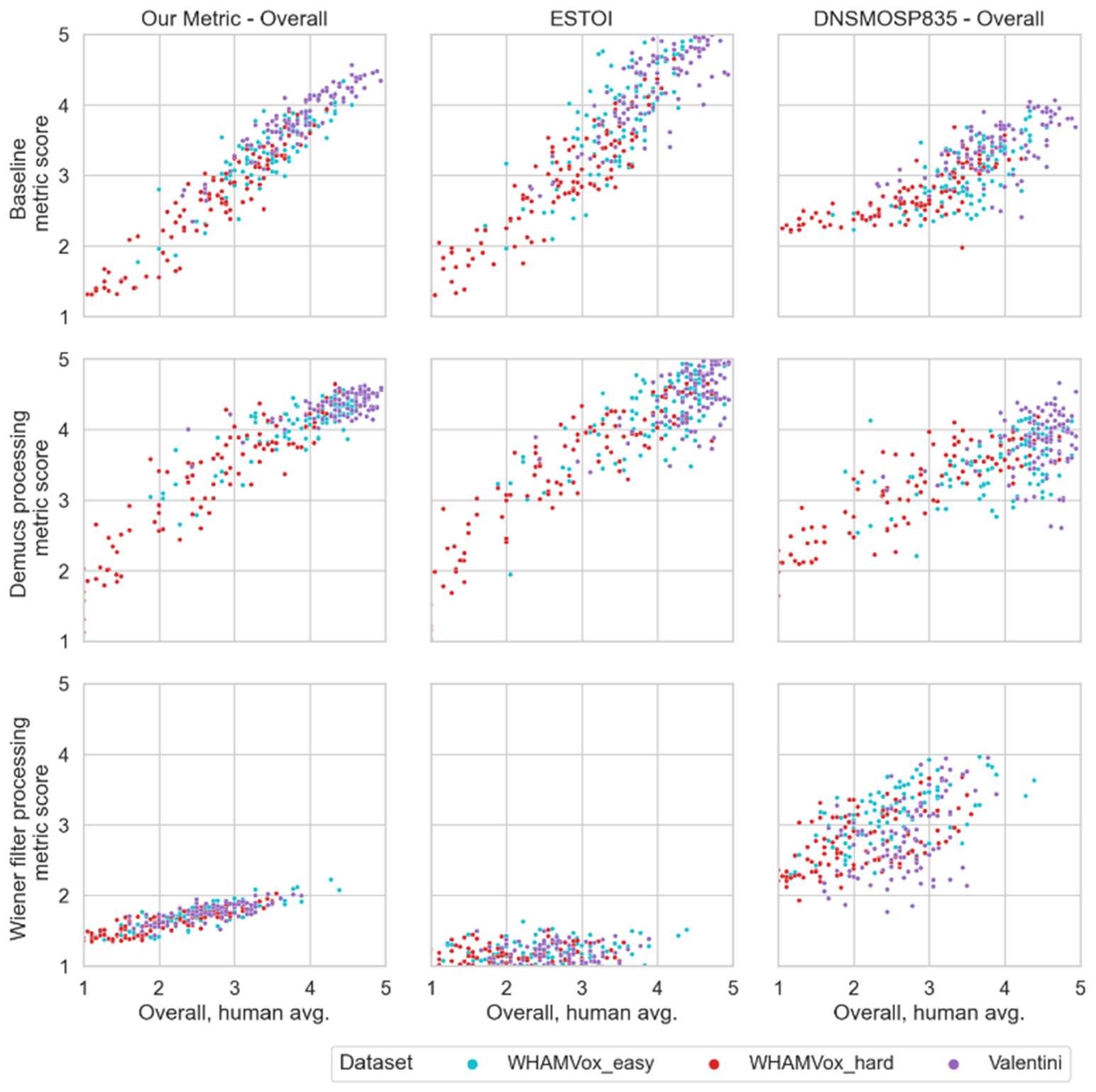


### Fig. A5. Comparison of our non-intrusive metric with ESTOI (intrusive) and DNSMOSP835 without 3^rd^ order correction on human ratings in overall quality, noise, speech quality.

*Web API usage*

The API itself is utilized using 2 endpoints: 1) the submit endpoint is used to submit audio files for evaluation 2) the result endpoint is used to retrieve the results for a submission. The evaluation is executed asynchronously. The submit endpoint returns a task ID which can be used to query the result endpoint. This result endpoint provides information about the status of a submission as well as the results of the submission once those are ready. To submit files, a POST request must be made to the submit endpoint. One or more audio files must be included in the request, for example:

curl -X 'POST' \

'https://metric.audatic.ai/api/submit/' \

-H 'accept: application/json' \

-H 'Content-Type: multipart/form-data' \

-F 'files=@0000.wav;type=audio/wav' \

-F 'files=@0001.wav;type=audio/wav'

The response from the API includes the generated task ID that will be used to retrieve the results:

{"task_id":"69e9959e-907f-49fa-9800-b19a5d08ead7"}

To retrieve the results, the user can query the result API endpoint using the task ID obtained while submitting the files:

curl -X 'GET' 'https://metric.audatic.ai/api/result/4b522bae-8e19-422e-89bc-ee00e8786b38'

Depending on the status of the submission, this endpoint will either return a response where status is PENDING or, if the submission is done, the results. For example:

{

"id":"69e9959e-907f-49fa-9800-b19a5d08ead7",

"status":"SUCCESS",

"error":null,

"result": {

"0000.wav":{

"overall":3.495769500732422,

"noise":3.350250005722046,

"sound_quality":3.982921838760376

},

"0006.wav":{

"overall":3.9978229999542236,

"noise":4.454800128936768,

"sound_quality":3.927513837814331

}

}

}

Our metric is trained using single-channel (mono) sound samples, and therefore the API will only accept single-channel audio files. Averaging stereo files can result in unwanted results, especially if the left and right channels differ considerably, and we want to encourage the users to actively think about the expected behavior of multi-channel files, e.g. are microphones close to each other or far away, are qualitative differences in the results expected or not. Each audio sample needs to be at least 4 seconds long, as this is the minimal input size for the metric. Samples can be up to 20 seconds long and resulting scores will be an average of all of the completed 4 second windows in the audio file. For example, if a 6-second-long audio file is submitted, the returned score will be for the 4 first seconds only. If an 8-second-long audio file is submitted, the returned score will the average of the predictions for seconds 0 to 4 and 4 to 8.
